# Cellular and Molecular Control of Lipid Metabolism in Idiopathic Pulmonary Fibrosis: Clinical Application of the Lysophosphatidic Acid Pathway

**DOI:** 10.3390/cells12040548

**Published:** 2023-02-08

**Authors:** Yusuke Nakamura, Yasuo Shimizu

**Affiliations:** Department of Pulmonary Medicine and Clinical Immunology, Dokkyo Medical University School of Medicine, 880 Kitakobayashi, Mibu 321-0293, Tochigi, Japan

**Keywords:** idiopathic pulmonary fibrosis, lipid metabolism, lysophosphatidic acid, induced pluripotent stem cells, S1P, pirfenidone, nintedanib

## Abstract

Idiopathic pulmonary fibrosis (IPF) is a representative disease that causes fibrosis of the lungs. Its pathogenesis is thought to be characterized by sustained injury to alveolar epithelial cells and the resultant abnormal tissue repair, but it has not been fully elucidated. IPF is currently difficult to cure and is known to follow a chronic progressive course, with the patient’s survival period estimated at about three years. The disease occasionally exacerbates acutely, leading to a fatal outcome. In recent years, it has become evident that lipid metabolism is involved in the fibrosis of lungs, and various reports have been made at the cellular level as well as at the organic level. The balance among eicosanoids, sphingolipids, and lipid composition has been reported to be involved in fibrosis, with particularly close attention being paid to a bioactive lipid “lysophosphatidic acid (LPA)” and its pathway. LPA signals are found in a wide variety of cells, including alveolar epithelial cells, vascular endothelial cells, and fibroblasts, and have been reported to intensify pulmonary fibrosis via LPA receptors. For instance, in alveolar epithelial cells, LPA signals reportedly induce mitochondrial dysfunction, leading to epithelial damage, or induce the transcription of profibrotic cytokines. Based on these mechanisms, LPA receptor inhibitors and the metabolic enzymes involved in LPA formation are now considered targets for developing novel means of IPF treatment. Advances in basic research on the relationships between fibrosis and lipid metabolism are opening the path to new therapies targeting lipid metabolism in the treatment of IPF.

## 1. Introduction

Idiopathic pulmonary fibrosis (IPF) is a disease with a poor prognosis, causing progressive fibrosis of the lungs. Two antifibrotic agents, i.e., pirfenidone (PFD) and nintedanib (NTD), are the only drugs available for clinical use in the treatment of this disease. These drugs are mainly effective in suppressing the disease progression but are poor in terms of the effect in alleviating the disease. As the pathogenesis of IPF has been increasingly elucidated in recent years, various therapeutic approaches have begun to be explored. Studies are now under way on numerous targets for IPF treatment, including autotaxin (ATX) [1], a metabolic enzyme involved in the lysophosphatidic acid (LPA) pathway; its receptor lysophosphatidic acid receptor 1 (LPA1) [2,3]; phosphodiesterase 4B (PDE4B), involved in the formation of profibrotic cytokines from monocytes and macrophages [4,5]; galectin-3, involved in profibrotic signal enhancement via various receptors [6]; connective tissue growth factor (CTGF), which induces fibrosis mediated by transforming growth factor-β (TGFβ) [7]; α_V_β_1_/α_V_β_6_ [8]; Janus kinase 2 (JAK2) [9]; Src tyrosine kinase [10], and so on. In addition, cell-based therapy using mesenchymal stem cells (MSCs) [11,12], adipose-derived MSCs [13,14], induced pluripotent stem cells (iPSC) [15,16], alveolar epithelial type II cells (AECII) [17,18], and so on has recently been studied within the framework of regenerative medicine.

In recent years, many findings regarding lipid metabolism and pulmonary fibrosis have been reported. Lipid-targeted therapeutic strategies are novel and potentially effective in the treatment of IPF, which has been difficult to control with existing drugs. Among others, many interesting reports from basic experiments are available concerning the fibrosis related to LPA signaling, inviting much expectation of clinical application. In this review, we first provide an overview of IPF and discuss current therapies. Next, the involvement of lipid metabolism in the pathogenesis of IPF is described, and finally, clinical trials targeting LPA signaling are discussed.

## 2. Idiopathic Pulmonary Fibrosis

### 2.1. Outline

IPF is a chronic progressive respiratory disease classified as idiopathic interstitial pneumonia. Its prognosis is poor, with the medial survival of patients reported to be 3–5 years after diagnosis [19,20]. The annual incidence is reported to be 6.8–17.4/100,000, with the prevalence being 42.7–63 per 100,000 population [21,22]. It causes progressive interstitial fibrosis, and patients die of acute exacerbation, progressive respiratory failure, lung cancer, and so on [19,20,23].

As far as the etiology is concerned, several genetic abnormalities have been found in some cases of this disease (reported as “familial pulmonary fibrosis”), including abnormalities of SP-C gene [24], ABC-A3 gene [25], and telomere-related genes (TERT, TERC) [26]. However, the exact cause remains unclarified. Involvement of various environmental factors and genetic polymorphisms (e.g., mucin composition: MUC5B, MUC2) and diverse views have been reported about the pathogenesis of this disease [27].

IPF is the most frequent in the Interstitial Lung Diseases (ILD); idiopathic ILDs include nonspecific interstitial pneumonia (NSIP), pleuroparenchymal fibroelastosis (PPFE), cryptogenic organizing pneumonia (COP), acute fibrinous and organizing pneumonia (AFOP), acute interstitial pneumonia (AIPOP), and acute pneumonia in the lungs, fibroelastosis (PPFE), cryptogenic organizing pneumonia (COP), acute fibrinous and organizing pneumonia (AFOP), acute interstitial pneumonia (AIP), respiratory bronchiolitis interstitial lung disease (RBILD), desquamative interstitial pneumonia (DIP), lymphoid interstitial pneumonia (LIP), and others. Autoimmune-related ILDs, hypersensitivity pneumonitis (HP), radiation-related ILD, and sarcoidosis are also considered ILDs [28].

Recent ILD guidelines have defined progressive pulmonary fibrosis (PPF) as ILD with progressive fibrosis. PPF includes not only IPF but also all fibrosing ILD. PPF is defined as at least two of the following three criteria occurring within the past year: (i) Worsening respiratory symptoms. (ii) Physiological evidence of disease progression (a. Absolute decline in FVC > 5% predicted within 1 year; b. Absolute decline in DLCO > 10% predicted within 1 year). (iii) Radiological evidence of disease progression (a. Increased extent or severity of traction bronchiectasis and bronchiolectasis; b. New ground-glass opacity with traction bronchiectasis; c. New fine reticulation; d. Increased extent or increased coarseness of reticular abnormality; e. New or increased honeycombing; f. Increased lobar volume loss) [28]. Nintedanib is recommended for PPF [28].

At the tissue level, this disease begins with sustained injury to the alveolar epithelium and microvascular endothelium (e.g., injury due to smoking, air pollution, microaspiration, and occupational exposure), resulting in inflammation and abnormal wound healing in the stroma. Then, there occurs transformation of fibroblasts, pericytes, epithelial cells, vascular endothelial cells, and fibrocytes into myofibroblasts which play a central role in fibrosis. Fibroblastic foci are formed from myofibroblasts, followed by excessive extracellular matrix formation and lung tissue remodeling. As a result, hardening and hypoxia occur in the tissue, leading to enhanced profibrotic cytokine formation and the activation of myofibroblasts. Thus, fibrosis advances in a loop-like manner [23,29,30,31,32,33].

Fibrosis involves various cytokines, chemokines, and growth factors such as interleukin (IL)-1β, tumor necrosis factor-α (TNFα), CTGF, TGFβ, IL-13, platelet-derived growth factor (PDGF), and fibroblast growth factor (FGF) [34,35]. The alveolar epithelial cells and vascular endothelial/mesothelial cells which have been injured by chronic stimuli produce diverse cytokines, of which TGFβ plays a central role in fibrosis [29,36]. TGFβ is known to be produced by alveolar epithelial cells (AECs), endothelial cells, fibroblasts, myofibroblasts, macrophages, and neutrophils [37,38]. The TGFβ secreted into the extracellular matrix (ECM) and stored there forms a complex with latency-associated peptide (LAP) and latent TGF-β binding protein (LTBP) to yield latent form TGFβ. The latent form TGFβ is activated via integrin αv (αv) [39,40] and thus the activated TGFβ binds to TGFβRII, followed by dimerization with TGFβRI and subsequent phosphorylation to enable signal transduction. The binding of this ligand activates many different downstream pathways, leading to the expression of fibrosis-related genes and induction of injury of airway epithelial cells, their transformation into myofibroblasts, epithelial-mesenchymal transition, and extracellular matrix deposition in tissues [38,41].

### 2.2. Current Status of IPF Treatment

Steroids, which are most frequently used for anti-inflammatory therapy, are poor in their effects against IPF, and there are few reports endorsing the effectiveness of their use in combination with immunosuppressors. Thus, it has been recommended to avoid the use of steroids in the treatment of IPF [42,43]. For the treatment of this disease with a poor prognosis, only two drugs, i.e., pirfenidone (PFD) and nintedanib (NTD), are now available clinically [28]. The effects of these drugs are confined to the suppression of disease progression and are unlikely to improve the once-reduced lung function. This chapter will describe PFD and NTD currently available for use.

#### 2.2.1. Pirfenidone

##### Pharmacology

PFD is the first drug shown to alleviate the reduced respiratory function and to extend the survival of patients with IPF [44,45]. Through the suppression of TGFβ production (a major action mechanism) [46,47,48], PFD inhibits fibroblast-myofibroblast transformation and cell proliferation mediated by TGFβ1/SMAD3 signals [49], reduces the levels of tenascin-c and fibronectin (extracellular matrix proteins) [50], and reduces the formation of collagen-specific chaperone of heat shock protein (HSP) 47 involved in procollagen secretion [51]. PFD is additionally known to suppress fibrosis through affecting platelet-derived growth factor (PDGF) [52] and basic fibroblast growth factor (bFGF) [53]. Macrophages on the airway are known to produce diverse cytokines and chemokines, thus contributing to wound healing and inducing immune reactions [54]. PFD has been reported to suppress the inflammatory cytokines produced by macrophages (IL-1, TNFα, TGFβ, PDGF [52,55]) as well as MCP1 (a chemokine produced by macrophages) [56]. PFD is considered to manifest antifibrotic effects via these mechanisms.

##### Clinical Trials on Pirfenidone

The first clinical trial was conducted in Japan. When the clinical usefulness was evaluated in that trial, patients treated with PFD showed improvement in the 6 min exercise test (primary endpoint), accompanied by suppression of vital capacity (VC) reduction (the secondary endpoint). [44]. In the phase 3 clinical trial subsequently conducted in Japan, the reduction in vital capacity (VC) at Week 52 (the primary endpoint) was suppressed significantly by PFD, accompanied by an extension of the progression-free survival (PFS), evaluated as the secondary endpoint [57]. Later, similar clinical trials (CAPACITY 004 and 006 Studies) were conducted overseas [58]. In the CAPACITY 004 Study (NCT00287729), 435 patients were allocated at random to a high-dose PFD group, a low-dose PFD group, and a placebo group. In the CAPACITY 006 Study (NCT00287716), 344 patients were allocated at random to a high-dose PFD group and a placebo group. The primary endpoint in these trials was the absolute change in percent predicted forced vital capacity (FVC) at week 72. In the CAPACITY 004 Study, FVC reduction was suppressed significantly by this drug (high-dose group −8.0% vs. placebo group −12.4%, *p* = 0.001), whereas no significant inter-group difference in FVC reduction was noted in the CAPACITY 006 Study (*p* = 0.501, high-dose group −9.0% vs. placebo group −9.6%) [58].

Thereafter, the ASCEND Study (NCT01366209) was conducted, allocating 555 patients at random to a high-dose PFD group and a placebo group [45]. In that trial, the change in percent predicted forced vital capacity (%FVC) or death at week 52 was set as the primary end point. When patients showing 10% or more reduction in FVC were defined as “decreased FVC” cases, the number of patients satisfying this definition or leading to death was significantly smaller in the high-dose PFD group than in the placebo group (*p* < 0.001; 46 patients, 16.5% vs. 88 patients, 31.8%).

Later, pooled post hoc analyses of these three clinical trials were conducted, revealing that the number of cases showing 10% or more reduction in FVC or leading to death was reduced by 43.8% (95%CI 29.3–55.4%) in the PFD treatment group, accompanied by significant alleviation or improvement in the secondary endpoints, i.e., reduction in 6 min walking distance, PFS, and the San Diego Shortness of Breath Questionnaire (SOBQ) score [59]. On the basis of these results, the clinical use of PFD was approved.

Also, in the phase 2 trial designed to evaluate the effects of PFD on progressive fibrosing interstitial lung disease (PF-ILD) other than IPF, the potential of this drug in manifesting a certain efficacy was reported [60,61].

As adverse reactions to this drug, eruption (29.2%) and gastrointestinal symptoms, such as nausea (35.5%), anorexia (12.4%), dyspepsia (17.8%), and diarrhea (24.6%), were often noted [59].

#### 2.2.2. Nintedanib

##### Pharmacology

NTD was initially developed as a drug for the treatment of solid cancer. Later, it was found to suppress the proliferation of fibroblasts and began to be used for the treatment of IPF. This is a multi-tyrosine kinase inhibitor, with a major function estimated as inhibiting the receptors involved in fibroblast activation such as platelet-derived growth factor receptor (PDGFR) α and β, fibroblast growth factor receptor (FGFR) 1–3, and vascular endothelial growth factor receptor (VEGFR) 1–3. To put it concretely, this drug binds to the ATP binding pocket and thus inhibits phosphorylation, leading to the inhibition of downstream cascade signals such as phosphatidylinositol-4,5-bisphosphate 3-kinase (PI3K), mitogen-activated protein kinase 1/2 (MEK1/2), and protein kinase B (Akt) [62,63,64].

There is a report that the PDGFR and FGFR expression levels in fibroblasts are higher in IPF patients than in controls, suggesting that these receptors may serve as critical targets for IPF treatment [65]. NTD has been reported to reduce PDGFR activity, fibroblast proliferation, and the transformation of fibroblasts into myofibroblasts [64]. In a study of fibroblasts derived from IPF patients, fibroblast proliferation was stimulated by each of PDGF, VEGF, and FGF administered separately, and treatment with NTD resulted in the dose-dependent suppression of fibroblast proliferation [65]. NTD has additionally been reported to reduce collagen production by fibroblasts stimulated with TGFβ [65]. The cascade involved in this activity has been reported to include mitogen-activated protein (MAP) kinase, extracellular signal regulated kinase (ERK), protein tyrosine kinase, and c-Abelson (c-Abl) [65]. Here, the TGFβ-Smad2/3 cascade (often studied in connection with IPF) was not found to be involved [65].

In addition, inhibitory activity against Flt3 and Src family (Src, Lyn, and Lck) has also been reported. The drug did not inhibit the other tyrosine kinase receptors (EGFR, HER2, InsR, IGF-IR, CDK1,2,4) [63].

Src is important as a target of NTD. TGFβ activates Src kinase, stimulating the transformation of fibroblasts into myofibroblasts. The Src inhibitor AZD0530 (saracatinib) inhibited the TGFβ signals, thus suppressing the transformation into myofibroblasts and the induction of collagen or fibronectin expression [66]. Resembling that report, Src inhibition by NTD has the potential of manifesting antifibrotic activity via a similar mechanism.

##### Clinical Trials on Nintedanib

The first large-scale clinical trial on NTD was a phase 2, randomized, double-blind, placebo-controlled trial (TOMORROW Study: NCT00514683). In that trial, 432 patients with IPF were allocated at random to NTD 300, 200, 100, and 50 mg groups and a placebo group, with the primary endpoint set as percent FVC reduction at 12 months. FVC reduction was suppressed by 68.4% in the NTD 300 mg group compared to the placebo group, although this difference was not statistically significant (NTD 300 mg group 0.06 L/year vs. placebo group 0.19 L/year) [67]. Later, INPULSIS I Study (NCT01335464) and INPULSIS II (NCT01335477) Study were carried out with a similar design, enrolling 515 and 551 patients with IPF, respectively. FVC reduction differed significantly between the NTD group and the placebo group in both INPULSIS I Study (NTD: −114.7 mL/year, placebo −239.9 mL/year; *p* < 0.01) and INPULSIS II Study (NTD: −113.6 mL/year, Placebo −207.3 mL/year; *p* < 0.01) [68]. On the basis of these results, the clinical use of NTD was approved.

Clinical trials on NTD have also been conducted in patients with progressive fibrosing interstitial lung disease (PF-ILD) other than IPF patients. In the phase 3 INBUILD Study (NCT02999178), percent annual FVC reduction until week 52 of treatment (mL/year) was set as the primary endpoint and compared between the NTD group and the placebo group. It was −80.8 mL in the NTD group and −187.8 mL in the placebo group (*p* < 0.0001), thus endorsing the drug’s effectiveness on PF-ILD in addition to IPF [69].

Gastrointestinal symptoms, including diarrhea (61.5–63.2%), nausea (22.7–26.1%), and vomiting (12.9–10.3%) were often seen as adverse reactions to NTD [68].

#### 2.2.3. Combined Therapy

Combined PFD + NTD therapy was evaluated in INJOURNEY Study (NCT02579603). Of the 105 patients with IPF initially treated with NTD, 53 patients were additionally treated with PFD (the add-on PFD group) and 5 patients continued to receive uncombined NTD therapy. These two groups were evaluated for 12 weeks. The primary endpoint was the percentage of patients with on-treatment gastrointestinal adverse events from baseline to Week 12. The exploratory endpoints were the absolute and relative FVC changes from baseline to Week 12 and the rate of decline in FVC. The incidence of gastrointestinal adverse events was 69.8% (37/53) in the add-on PFD group and 52.9% (27/51) in the NTD alone group. The mean change in FVC at 12 weeks was −13.3 (S.E. 17.4) mL in the add-on PFD group and −40.9 (S.E. 31.4) mL in the NTD alone group. The combined use of PFD and NTD was shown to be tolerable and safe in this study and the other [70,71]. Further evaluation is needed about the efficacy of this combined therapy.

## 3. IPF and Lipid Metabolism

### 3.1. Outline

Numerous reports have recently been published concerning IPF and lipid metabolism, indicating that this is a field attracting close attention at present. This chapter will outline the lipid metabolism and pathogenesis related to IPF, excluding the lysophosphatidic acid (LPA) cascade.

### 3.2. Eicosanoids

**Figure 1:**
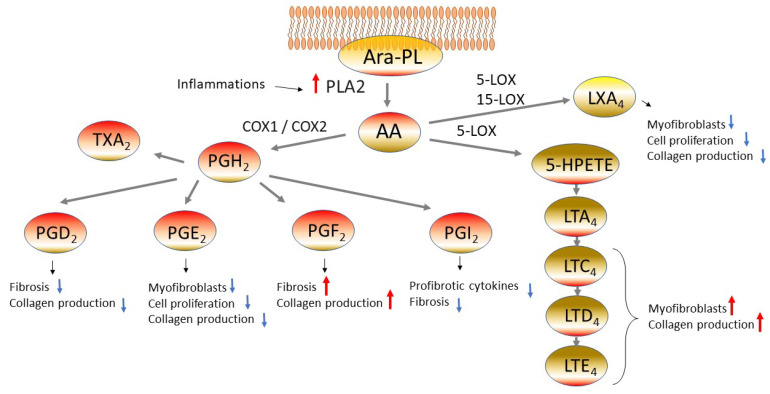
Eicosanoids pathway in fibrotic lung diseases. Arachidonic acid (AA) is freed from arachidonic acid esterified phospholipid (Ara-PL) by phospholipaseA2 (PLA2). AA is metabolized by cyclooxygenase-1 or -2 (COX1 or COX2) or by 5-lipoxygenase (5-LOX), yielding various eicosanoids. The eicosanoids manifest diverse physiological actions related to the pathogenesis of pulmonary fibrosis. Abbreviations: prostaglandin D2 (PGD2), prostaglandin H2 (PGH2), prostaglandin F2 (PGF2), prostaglandin I2 (PGI2, prostacyclin), thromboxane A2 (TXA2), 5-hydroxyeicosatetraenoic acids (5-HETE), leukotriene A4 (LTA4), leukotriene C4 (LTC4), leukotriene D4 (LTD4), leukotriene E4 (LTE4), lipoxin A4 (LXA4).

Eicosanoids, which are metabolites of arachidonic acid, are also reported to be involved in fibrosis. Arachidonic acid esterified phospholipid (Ara-PL) undergoes cleavage of arachidonic acid (AA) by phospholipase A2 (PLA2) induced by various stimuli. Thus, the freed AA is converted into various eicosanoids by cyclooxygenase-1 or -2 (COX1 or COX2) or by 5-lipoxygenase (5-LOX) (Figure 1) [72]. This section will describe the mechanism for pulmonary fibrosis involving various eicosanoids.

#### 3.2.1. Prostaglandin E_2_

The cells derived from IPF patients have been reported to show reduced levels of cyclooxygenase-2 (COX-2) and prostaglandin E_2_ (PGE_2_), leading to the long-lasting view that eicosanoids are involved in the pathogenesis of IPF [73,74]. It has also been reported that the expression of prostaglandin E synthetase (PTGES), an enzyme involved in PGE_2_ synthesis, is low in the lungs of IPF patients [75]. PGE_2_ is one of the lipids having been actively studied as a lipid with antifibrotic activity. PGE_2_ has been reported to suppress the proliferation of fibroblasts and their transformation into myofibroblasts and to reduce collagen production [74,76]. In a mouse model of bleomycin-induced fibrosis, PGE_2_ was suggested to work protectively against fibrosis [77]. The bronchoalveolar lavage fluid (BALF) after a bleomycin dose showed elevation of the PGE_2_ level, suggesting the possibility that PGE_2_ works in physiological defensive reactions. When PGE_2_ was administered in advance to bleomycin in an animal model of bleomycin-induced interstitial pneumonia, the collagen level and the histological fibrosis score (Ashcroft score) on 21 days after the dose showed improvement, while administration of PGE_2_ after the bleomycin dose failed to manifest therapeutic efficacy [77]. These results suggest that PGE_2_ has physiological antifibrotic activity. In the lungs, PGE_2_ is produced by epithelial cells and fibroblasts [78], and a study using IPF-derived fibroblasts showed less suppression of collagen synthesis and cell proliferation by PGE_2_ [79]. There is also a report that the stiffened matrix arising from fibrosis reduces PGE_2_ synthesis [75]. Thus, PGE_2_ is considered to manifest protective activity during the early phase of fibrosis and can serve as a critical target of IPF treatment.

#### 3.2.2. Cysteinyl Leukotrienes

The senescent cells, which are unable to proliferate as a result of aging, are considered one of the significant etiological factors for IPF, and it has been reported that IPF can be characterized by an increase of alveolar epithelial type II cells (AECII) which are p53 and p21^WAF^ (senescent cell markers) positive [80]. Lipid composition in senescent cells has also been studied, revealing elevated levels of 5-lipoxygenase (5-LOX), *LTC4S*, and *LTA4H* in human fibroblasts and elevation of cysteinyl leukotrienes (cysLTs) in conditioned medium (CM) [81]. The senescent fibroblasts prepared from IPF patient-derived cells showed increased expression of arachidonate 5-lipoxygenase (*ALOX5)* but no elevation of the enzymes involved in the synthesis of prostaglandins such as *PTGS2* (COX-2)*, PTGDS*, and *PTGES* [81]. It is probable that the lipoxygenase-inducing cascade becomes dominant during the processes of arachidonic acid metabolism in the presence of IPF. When the cysLTs-rich CM collected from senescent fibroblasts was administered to non-senescent fibroblasts, the expression of *COL1A2* and *αSMA* increased, and this change was inhibited by a 5-LOX inhibitor (zileuton) but not by a COX-2 inhibitor (NS-398) [81]. These results suggest the involvement of cysLTs in the pathogenesis of pulmonary fibrosis. Furthermore, the conditioned medium derived from senescent cells induced *COL1A2* expression in fibroblasts, while montelukast, known to inhibit cysteinyl leukotriene receptors 1 (CysLT1), suppressed *COL1A2* [81,82]. There are multiple other reports suggesting that montelukast can serve as a target of fibroblast treatment [83,84], providing a perspective for its clinical application in IPF management in the future.

#### 3.2.3. Lipoxin A_4_

Similar to LTs, lipoxin A_4_ (LXA_4_) is an eicosanoid metabolized by 5-LOX and 15-lipoxygenase (15-LOX). LXA_4_ has been shown to suppress TGFβ1-dependent collagen secretion, αSMA expression, and cell proliferation in fibroblasts derived from IPF patients. It is desirable that LXA_4_ is further studied from now on [85].

#### 3.2.4. Prostaglandin I_2_

Of the other eicosanoids, prostaglandin I_2_ (PGI_2_; prostacyclin) has also been reported to be antifibrogenic. Iloprost, a prostacyclin analogue, has been reported to have suppressed profibrotic cytokines (TNFα, IL-6, and TGFβ1) and fibrosis in a mouse model of bleomycin-induced fibrosis [86].

#### 3.2.5. Prostaglandin F_2α_

Prostaglandin F_2α_ (PGF_2α_) is considered to be profibrogenic. The level of PGF_2α_ in the BALF was higher in IPF patients than in the disease control group [87], and this prostaglandin was shown to stimulate fibroblast proliferation and collagen production in a manner independent from TGFβ [87]. In mice lacking the PGF_2α_ receptor “prostaglandin F (PGF) receptor (FP)” (*Ptgfr^−/−^*), bleomycin-induced pulmonary fibrosis was suppressed [87]. Thus, PGF_2α_ is involved in the pathogenesis of pulmonary fibrosis and can serve as a target for treatment of pulmonary fibrosis.

#### 3.2.6. Prostaglandin D_2_

Prostaglandin D_2_ (PGD_2_) is catalyzed by hematopoietic prostaglandin D synthase in the presence of glutathione and produced primarily in activated mast cells and T cells, and has a suppressive effect on fibrosis [88,89]. The treatment of human fibroblasts with PGD_2_ resulted in c-AMP-mediated suppression of TGFβ-induced type I collagen secretion. A similar suppressive activity has been observed also with BW245C, a PGD_2_ receptor (DP-receptor: DP1) agonist, thus suggesting the involvement of PGD_2_-DP1 signals in suppression of fibrosis [90]. Another PGD_2_ receptor “chemoattractant receptor homologous with T-helper cell type 2 cells (CRTH2)” is also considered to be involved in the suppression of fibrosis. CRTH2 is expressed in type 2 cytokine-producing T cells and in group 2 innate lymphoid cells (ILC2). CRTH2 deficient mice (CRTH2^−/−^) showed aggravation of bleomycin-indued fibrosis as well as elevation of inflammatory cells in BALF and elevation of lung tissue *Col1a1*. The transplant of splenic δγT cells into CRTH2^−/−^ mice resulted in the suppression of fibrosis, suggesting that CRTH2 in these cells affects fibrosis [91].

### 3.3. Sphingolipids

**Figure 2:**
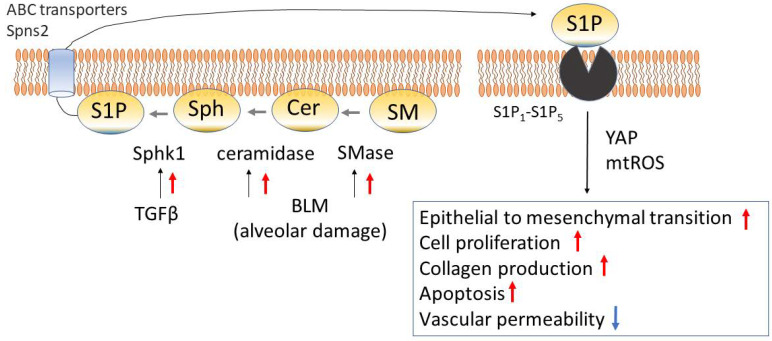
Sphingolipids pathway in fibrotic lung diseases. Sphingomyelin (SM) is metabolized by sphingomyelinase (SMase), yielding ceramide (Cer). It is later converted by ceramidase into sphingosine (Sph) and subsequently converted by sphingosine kinase 1 (Sphk1) into sphingosine-1-phosphate (S1P). S1P communicates with the inside and outside of cells via ATP-binding cassette (ABC) transporters and spinster homolog 2 (Spns2) and binds to S1P receptors to manifest physiological activity. Abbreviations: Bleomycin (BLM), Hippo/yes-associated protein (YAP), mitochondrial reactive oxygen species (mtROS).

The cell membrane is composed of lipids such as phospholipids and sphingolipids. These lipids work not only as cell membrane components but also as factors with physiological activity of transducing diverse signals in response to various stimuli [92]. Glycerophospholipid will be discussed later, and this paragraph focuses on sphingolipids. Sphingomyelin (SM) is a representative of sphingolipids found in cell membranes, assuming the form of sphingophospholipid composed of phosphocholine (PC) or phosphoethanolamine (PE) bound to the ceramide (Cer). Sphingomyelinase (SMase) is formed by various external factors, and Cer is formed as a result of hydrolysis. Cer is converted into sphingosine (Sph) by ceramidase. Then, under the catalytic activity of sphingosine kinase 1 (Sphk1) distributed in the cell membrane, sphingosine-1-phosphate (S1P) is formed [93]. S1P is released from cells mediated by ATP-binding cassette (ABC) transporters [94] or spinster homolog 2 (Spns2) [95]. Five subtypes of S1P receptor (S1P_1_–S1P_5_) are known, all assuming the form of seven-transmembrane trimer type G protein-coupled receptor (Figure 2). Niemann–Pick disease types A and B are caused by SM accumulation due to a shortage of acid sphingomyelinase (ASM), and these diseases occasionally induce pulmonary fibrosis [96]. Sphk1 is induced by TGFβ, a cytokine playing a central role in fibrosis [97].

When studied in mice, administration of bleomycin elevated the lung tissue ASM activity, accompanied by elevation in Cer and acid ceramidase (AC) activity. In ASM^−/−^ mice, collagen production was suppressed, accompanied by a reduction of the apoptosis rate and suppression of transformation into myofibroblasts. When ASM in NIH3T3 fibroblasts was blocked with small interfering (si) RNA, a reduction in sphingosine-1-phosphate (S1P) levels was induced and collagen production was suppressed [98]. This result suggests the possible influence of S1P on fibrosis and indicates that activation of each synthetase in the presence of fibrosis-inducing stimuli will lead to S1P production.

When human fibroblasts were stimulated with TGFβ or bleomycin, the levels of mitochondrial reactive oxygen species (mtROS) and transcription factors, such as Hippo/yes-associated protein (YAP), fibronectin (FN), and αSMA, were elevated. Inhibition of Sphk1 or YAP resulted in a decrease of mtROS and less expression of FN and αSMA. Furthermore, inhibition of each of S1P and YAP resulted in a similar decrease of mtROS and less expression of FN and αSMA [99]. On the basis of these findings, S1P is considered to possibly induce mitochondrial damage and YAP-mediated fibrosis.

There is a report demonstrating a significant elevation of the S1P level in the serum and BALF of IPF patients. S1P has additionally been reported to have roles in epithelial-mesenchymal transition (EMT) in stimulated-AECII, cell proliferation, and collagen production [100].

Regarding the activity of S1P on vascular endothelial cells, the effects in reducing vascular permeability and the protective activity are known [101]. The elevation of vascular permeability is involved in the pathophysiology of pulmonary fibrosis at the acute stage [102]. Mice with endothelial cell-(EC) specific deletion of the S1P receptor 1 (S1PR1) (EC-S1pr1^−/−^) showed enhanced permeability across the pulmonary vessels. Intratracheal administration of bleomycin to EC-S1pr1^−/−^ mice resulted in enhanced pulmonary vascular permeability and aggravation of fibrosis compared to wild-type mice [102]. It seems possible that the S1P-S1PR1 pathway in vascular endothelial cells induced immune cell infiltration and activation of the coagulation via enhanced pulmonary vascular permeability, leading to aggravation of fibrosis [102].

A sphingosine analogue, FTY720 (Fingolimod) is a drug known to be phosphorylated by sphingosine kinase 2 and to function as an S1P analogue [103]. It has already been introduced clinically as a drug for the treatment of multiple sclerosis. In an animal model of bleomycin-induced interstitial pneumonia, low doses of FTY720 during inflammatory phases could suppress fibrosis, while such doses during remodeling phases had the potential of aggravating the fibrosis [104]. S1P may work protectively if used during the inflammatory phases of acute vascular permeability reduction. On the other hand, if used during the fibrotic phases, it can stimulate fibroblast proliferation and collagen production, leading to aggravation of fibrosis. More detailed fundamental evaluation is desirable before clinical application of S1P to the management of pulmonary fibrosis.

### 3.4. Lipid Balance and Fibrosis

Elongation of long-chain fatty acid family member 6 (Elovl6), a kind of fatty acid elongation, has been reported to aggravate bleomycin-induced pulmonary fibrosis through disturbing the balance in fatty acid composition [105]. Elovl6 is a rate-limiting enzyme for the elongation of saturated and monounsaturated long chain fatty acids and is known to catalyze the elongation of palmitate (PA: C16:0) to stearate (C18:0). Stearate is converted by stearoyl CoA desaturase (SCD) into oleic acid (OA; C18:1 n-9). If the expression of Elovl6 is reduced by bleomycin, the PA level rises, leading to elevation of reactive oxygen species (ROS), eventually resulting in the progression of fibrosis mediated by TGFβ elevation and apoptosis [105]. This has additionally been shown to induce apoptosis in a study of PA at the cellular level [106]. That is a significant report indicating that changes in fatty acids are involved in fibrosis. Therefore, measurement and adjustment of lipid profiles in the living body may serve as a therapeutic strategy.

Phospholipid profiles in the serum of IPF patients have also been measured. Analysis of the serum lipids in IPF patients by ultra-high performance liquid chromatography coupled to high-resolution mass spectrometry (UHPLCHRMS) revealed elevated levels of lysophosphatidylcholine (LPC) [107]. That report referred also to the usefulness of LPA (lysophosphatidic acid) as a biomarker, and there are also reports demonstrating elevated LPA levels in the BALF and exhaled breath condensate from IPF patients [108,109]. Therefore, the above-mentioned elevation in the LPC level may reflect the event during the course of LPC metabolism into LPA. Elevation in the LPA level has also been reported for patients with other diseases such as bronchial asthma [110], hyperoxic lung injury [111], and COPD [112]. Another study measured 507 individual serum circulating lipid profile of IPF by quadrupole time of flight mass spectrometry (UPLC-QTOF/MS) with lipidomics, and six discriminating lipids were selected, such as stigmasteryl ester, dodecatrienyl dodecanoate, deoxyvitamin D3, triacylglycerol, and diacylglycerol, as the potential biomarkers in diagnosing IPF [113].

Lipid composition has also been studied in the IPF-affected lung tissue, revealing the elevation of palmitic acid (16:0) and stearic acid (18:0) [114]. In a study of a mouse model of bleomycin-induced interstitial pneumonia, the mice fed with a palmitic acid-rich high-fat diet showed exacerbation of fibrosis and decreased survival rate, accompanied by elevation in the TGFβ level [114]. In the epithelial cells, treatment with palmitic acid (16:0) induced increases in endoplasmic reticulum (ER) stress and apoptosis [114]. In mouse lung epithelial cells (MLE12 cells), inhibition of CD36 (a fatty acid transporter) reduced the effect of palmitic acid (16:0) on cell viability and caused decreases in profibrotic cytokines (MCP-1 and IL-6), ER stress markers, and apoptosis markers [114]. That study indicates the possibility that oral intake of palmitic acid serves as a factor responsible for the pathogenesis of IPF, and the data from that study are valuable. If the details of ingested foods are analyzed in relation to pulmonary fibrosis in clinical cases, a new approach for the treatment of pulmonary fibrosis may be cultivated.

The role of other lipids has also been investigated in preclinical studies [115]. Exposure to high-fat diet can be a trigger of lung fibrosis by neutrophilic inflammation or TGFβ stimulation [116,117]. Docosahexaenoic acid (DHA) is a ω-3 polyunsaturated fatty acid (PUFA), and this lipid has reported to attenuate lung fibrosis by upregulation of Smad7 [118]. Another PUFA of gamma-linolenic acid (GLA) has also attenuated fibrosis. In hamster bleomycin-induced fibrosis model, GLA dietary intake leads to elevation of prostaglandin E1 (PGE1) which is metabolite of PGE2 and has anti-inflammatory effect, then showed attenuate lung inflammation and fibrosis [118].

### 3.5. Statins

Statins are extensively used for the treatment of hypercholesterolemia during clinical practice. They have recently been shown to have anti-inflammatory activity as well. In a mouse model of bleomycin-induced fibrosis, treatment with pravastatin suppressed the expression of TGFβ, CTGF, and RhoA as well as the expression of a fibrosis marker, hydroxyproline [119]. In recent studies, the ACEII and fibroblasts of patients with pulmonary fibrosis were shown to have markedly low levels of low-density lipoprotein receptor (LDLR). In Ldlr deficient (Ldlr^−/−^) mice, the level of low-density lipoprotein (LDL) was markedly high, and the administration of bleomycin resulted in the rapid onset of intense fibrosis. LDL was thus estimated as a factor responsible for the induction of apoptosis and TGFβ [120]. Also, clinically, there is a report that the use of statins (drugs for the treatment of hypercholesterolemia) in IPF patients reduced the hospitalization rate and the death rate due to IPF [121].

### 3.6. Involvement of Mitochondria

There is a report that mitochondrial dysfunction in type II alveolar epithelial cells exacerbated fibrosis [122]. In that report, increased mitochondrial injury was noted in IPF patients. When airway epithelial cells were studied in vitro, stimulation with TGFβ resulted in signs of mitochondrial depolarization and mitochondrial ROS (reactive oxygens species), thus indicating that mitochondrial injury was caused. Mitochondrial injury is known to stimulate apoptosis and profibrotic cytokines, leading to pulmonary fibrosis [122,123]. As stated above, the appearance of mitochondrial ROS (mtROS) possibly leading to mitochondrial injury can also be induced from S1P signals [99].

We previously reported that the phosphatidylserine decarboxylase (PISD), an enzyme for lipid metabolism found in mitochondria, was decreased by TGFβ, leading to a change in the phospholipid composition. In fibroblasts, knocking down the PISD resulted in the elevation of αSMA and collagen production, suggesting that PISD may be contributing to the mechanism for fibrosis through the adjustment of phospholipids [124].

### 3.7. Lipofibroblast

Myofibroblasts are an important source for collagen production. Metformin, a drug for the treatment of diabetes mellitus, has been reported to have the potential of inducing the transformation of myofibroblasts into lipid droplet-containing interstitial fibroblasts (lipofibroblasts) mediated by AMP-activated protein kinase (AMPK). COL1A1 expression is lower in lipofibroblasts than in myofibroblasts, and the effect in maintaining AECII (known to be involved in lung regeneration) is expected of lipofibroblasts. Thus, lipofibroblasts are promising as a target for the treatment of pulmonary fibrosis from now on [125,126].

### 3.8. Points for Consideration in Connection with Regenerative Medicine

As illustrated above, lipid metabolism is involved in the pathogenesis of fibrosis. Novel treatment methods are being developed as mentioned above, including the evaluation of cell-based therapy. Cell-based therapy is an approach of regenerative medicine expected to manifest anti-inflammatory activity through the administration of stem cells. When regenerative medicine is considered, it is also essential to evaluate the lipid composition in each product used for regenerative medicine [127]. Suppression of fibrosis by means of stem cell administration has been attempted with mesenchymal stem cells (MSCs) [11,12], adipose-derived MSCs [13,14], induced pluripotent stem cells (iPSC) [15,16], and AECII [17,18]. Stem cell-based treatment is still challenging; however, MSC therapy in IPF has shown beneficial results [128]. Because the lipid composition in stem cells is altered by the differentiation of stem cells [129], evaluation of lipid composition is essential in evaluation of the quality of regenerative medicine products. It also needs to be taken into consideration that the altered lipid profile can give rise to profibrotic lipid mediators.

The relationship between vascular meshwork and fibrosis during IPF pathogenesis has been actively analyzed, yielding reports that lung tissue fibrosis is affected by the stimulation of fibroblasts mediated by capillary endothelial cells [130] and that myofibroblasts produce neovascularization suppressive factors, leading to suppressed neovascularization [131]. There is also a view that the reduction in vascular meshwork causes abnormal repair of tissues, possibly leading to fibrosis [132]. It is important to analyze the lipid profile of vascular endothelial cells which constitute blood vessels and to obtain basic data. Our study revealed elevation of plasmalogen phosphatidylethanolamines (38:5) and (38:4) during the course of differentiation from iPSC to vascular endothelial cells [133]. It was additionally shown that lysophosphatidylcholine (LPC) (22:5) was predominantly detected during the course of network formation by iPSC-derived vascular endothelial cells and that their distribution was not uniform [134].

When regenerative medicine is considered, it needs to be borne in mind that the lipid composition is likely to change depending on the conditions and that attention is needed to be paid not only to the composition but also to the distribution of lipids.

## 4. LPA Involvement in IPF and Its Pathogenesis

### 4.1. Outline

The LPA cascade has been attracting close attention in recent years because it has the potential of explaining the pathogenesis of IPF and being utilized for therapeutic strategy. The BALF of IPF patients is known to be significantly rich in LPA [108], and LPA has been studied for analyzing the pathogenesis of IPF and as a target of IPF treatment. LPA receptors assume the form of G protein-coupled receptors (GPCRs). Six subtypes are known, and LPA1 and LPA2 are considered to be primarily involved in IPF [135]. This section will outline LPA and describe the clinical studies conducted to date.

### 4.2. Pathways for LPA Metabolism

**Figure 3:**
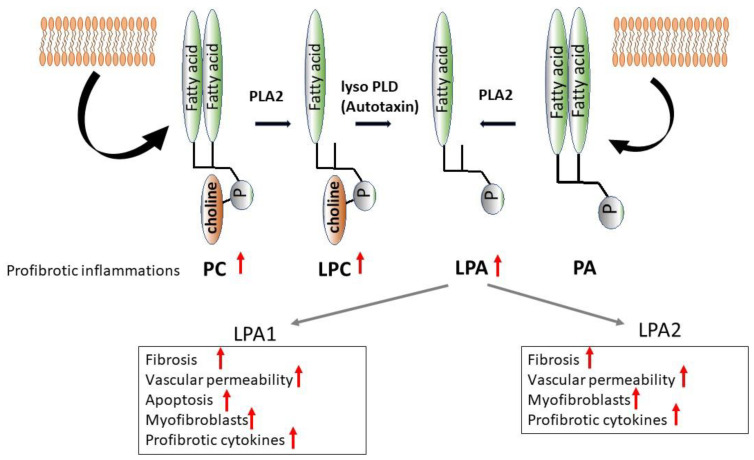
LPA pathway in fibrotic lung diseases. A representative pathway for lysophosphatidic acid (LPA) production is shown here. Glycerophospholipids, such as phosphatidylcholine (PC) and phosphatidylethanolamine (PE), are converted by phospholipase A2 (PLA2) into lysophosphatidylcholine (LPC). LPC is then converted by lysophospholipase D (lysoPLD: Autotaxin: ATX) into LPA. Another pathway involves the formation of lysoPLD/ATX from phosphatidic acid (PA). Later, the signals are transduced to the LPA receptors (LPA1/LPA2), resulting in the manifestation of physiological activity related to pulmonary fibrosis.

The pathway for LPA metabolism is diverse [136], and a representative pathway is shown below. The formation of LPA begins with the metabolism of glycerophospholipids (GPs) such as phosphatidylcholine (PC) and phosphatidylethanolamine (PE) abundantly found in the cell membranes. Dipalmitoylphosphatidylcholine (DPPC) is a major phospholipid found in the alveoli, and here PC is taken as an example for further discussion. PC is degraded by phospholipase A2 or phospholipase A1 (PLA2 or PLA1) into lysophosphatidylcholine (LPC) [137] and LPC is converted by lysophospholipase D (lysoPLD: Autotaxin/ATX) into lysophosphatidic acid (LPA) [138]. In addition, phosphatidic acid (PA) is formed by metabolism with lysoPLD/ATX (Figure 3).

Using an animal model of bleomycin-induced interstitial pneumonia, we analyzed the profiles of PC, PE, and LPC in the serum and BALF. The levels of PC, PE, and LPC in the BALF were found to be significantly elevated for many fatty-acid-binding patterns (unpublished data). These results suggest the possibility that phospholipids, such as PC and PE, were released locally in the alveolar epithelium and underwent metabolism into lysophospholipids. If this finding is combined with the past report that the lung tissue’s chromatic response to ATX was elevated in IPF patients [139], it seems likely that the metabolism of lysophospholipids into LPA occurs locally, contributing to the pathogenesis of interstitial lung disease.

### 4.3. LPA Signal Transduction and Mechanism for Fibrosis

Resembling the migration of fibroblasts to a certain site of the fibrin matrix to form granuloma there during the course of skin injury, it is known that fibroblasts accumulate in the fibrin-rich exudate from airspaces during the course of IPF [140]. The BALF from IPF patients has been reported to increase the chemotaxis of pulmonary fibroblasts significantly [141,142], and this activity seems to be involved in the pathogenesis of IPF. Data are available concerning the correlation between such chemotactic response of fibroblasts and lung function [141], allowing an expectation to use it as an important target of treatment. Subsequent studies revealed that LPA is a substance involved in this response, thus triggering research into the involvement of LPA in IPF.

#### 4.3.1. LPA1

Stimulation of fibroblast’s chemotaxis by LPA was inhibited by an LPA1 inhibitor, Ki16425 [108], indicating that the LPA/LPA1 signals are important for chemotaxis. In LPA1 knockout (KO) mice, bleomycin-induced fibrosis was reduced and the hydroxyproline content in the lungs decreased significantly, accompanied by a significant improvement of the 21-day survival rate [108]. There are data indicating that LPA1 receptors are significantly predominant among the LPA receptors in IPF patients [107], suggesting the possibility for LPA1 to serve as the target for treatment. There is also a report that LPA/LPA1 signals affected the vascular endothelial cells, resulting in exacerbation of the vascular permeability in LPA1 KO mice [108].

The LPA/LPA1 cascade also affects the epithelial cells. Apoptosis of epithelial cells is considered to be involved in the onset of IPF [143]. LPA1 KO mice showed significantly less apoptosis of alveolar epithelial cells. Apoptosis of bronchial epithelial cells was also suppressed in these mice. Of the LPA species, LPA (16:0) and LPA (18:0) increased soon (Day 1) after bleomycin treatment, followed 3 days later by an increase of LPA(18:1), LPA(18:2), and LPA(20:4) [144]. In kidney epithelial cells, stimulation of EMT (epithelial-mesenchymal transition) via the LPA1/MAPK-AKT/KLF5 signaling pathway has been reported, and a similar event may be occurring also in the lungs [145]. The LPA1/3 antagonist VPC12249 has been reported to have attenuated the pulmonary fibrosis in a mouse model of radiation lung fibrosis. Following the finding of enhanced LPA1/3 expression in mice induced by 16 Gy radiation, VPC12249 was administered to these mice, resulting in the suppression of survival shortening and pulmonary fibrosis and decrease of profibrotic cytokines such as TGFβ and CTGF. That study is interesting in that the LPA1/3 signals stimulated the expression of CTGF. An anti-CTGF antibody (pamrevlumab) is now under clinical trial (currently at phase 3) as a novel drug for IPF treatment [7].

#### 4.3.2. LPA2

Evaluation has also been made on LPA2. LPA2 KO (Lpar2^−/−^) mice, studied as a model of bleomycin-induced interstitial pneumonia, showed suppressed expression of fibronectin and αSMA, accompanied by decreases in lung tissue collagen and pulmonary vascular leakage and improvement of the survival rate [146]. Furthermore, the levels of profibrotic cytokines (IL-6, TGFβ) in BALF decreased. Also, when human fibroblasts were inhibited with small interfering siRNA for LPA2, the expression of fibronectin, Col1A2, αSMA, and TGFβ was decreased [146]. A possible mechanism for this change is the influence of LPA2 signals on the extracellular regulated kinase (ERK)1/2, Akt, Smad3, and p38 mitogen-activated protein kinase (p38) cascade [146]. Interesting enough, LPA2 was shown to have the potential of enhancing the TGFβ/Smad2/3 signals [146].

There is also a report showing that in epithelial cells, LPA activates TGFβ mediated by αvβ6 integrin in the LPA2-RhoA/Rho kinase cascade [147]. In that study, data showing increases of LPA2 and αvβ6 integrin in IPF patients were also collected, suggesting the possibility of clinically applying LPA2 as a target of treatment [147].

#### 4.3.3. ATX

ATX (autotaxin), i.e., ectonucleotide pyrophosphatase/phosphodiesterase family member 2 (Enpp2), is an enzyme involved in LPA formation and has been reported to be expressed abundantly in the alveolar epithelium, fibroblastic foci, and alveolar macrophages of IPF patients [139] (Figure 3). It has been studied also in an animal model of bleomycin-induced interstitial pneumonia, revealing significant elevation of the ATX level in BALF. ATX^−/−^ animals died at embryonic day 10.5 because of vascular hypoplasia [148]. For this reason, conditionally knockout animals are used for the evaluation of ATX after maturation. Mice with conditionally knockout bronchiolar epithelial cells or alveolar macrophages were created to evaluate ATX deficiency. Although some parameters did not differ significantly between these two groups of knockout mice, ATX conditionally knockout mice showed a reduction in lung collagen, total cell counts in BALFs, BALF soluble collagen concentrations, BALF ATX concentrations, and ATX activity. These results suggest that bronchiolar epithelial cells and alveolar macrophages are contributing at least to the formation of ATX in lungs [139]. In the same study, it was additionally shown that treatment with the ATX inhibitor GWJ-A-23 alleviated fibrosis in the mouse model of bleomycin-induced interstitial pneumonia [139].

### 4.4. Therapeutic Strategy Focusing on the LPA Cascade

As described above, LPA1 antagonists and ATX inhibitors deserve evaluation as targets of IPF treatment. Table 1 lists the drugs under clinical studies revealed by the literature search via U.S. National Library of Medicine, ClinicalTrials.gov (Table 1). This chapter will outline the candidate drugs currently evaluated for use in treatment.

#### 4.4.1. LPA1 Antagonist

Clinical studies on LPA1 antagonists are now under way on BMS-986020 and BMS-986278.

##### BMS-986020

IM136003 (NCT01766817) is a phase 2 clinical trial on the use of BMS-986020 for IPF. Between April 2013 and February 2016, 373 participants were enrolled to the trial and 143 of them were randomized to three groups (placebo group, n = 47; BMS-986020 600 mg quaque die (qd) group, n = 48; BMS-986020 600 mg bis in die (bid) group, n = 48) for this phase 2, multicenter, randomized, double-blind trial involving patients with IPF having FVC 45–90% and diffusing capacity for carbon monoxide (DLCO) 30–80%. The primary endpoint was the change from baseline in FVC rate to Week 26. FVC reduction was significantly smaller in the BMS-986020 600 mg bid group than in the control group (−0.134 L vs. −0.042 L, *p* = 0.049) [2]. In the BMS-986020 600 mg bid group, three patients prematurely quit the study because of treatment-related cholecystitis that resulted in cholecystectomy [2]. No case of such hepatic impairment was noted in the phase 1 trial in which the subjects were confined to healthy individuals and the dosing period was short. Furthermore, alanine aminotransferase (ALT) elevation (≥3× upper limit of normal) arose at an incidence of 7% (n = 3/48) in the BMS-986020 600 mg qd group, 21% (n = 10/48) in the 600 mg bid group, and 0% (0/47) in the placebo group. The incidence of elevation in other enzymes was as follows: aspartate aminotransferase (AST) placebo 0% vs. 600 mg qd 4% vs. 600 mg bid 6%, alkaline phosphatase (ALP) placebo 0% vs. 600 mg qd 0% vs. 600 mg bid 4%, and total bilirubin placebo 0% vs. 600 mg qd 4% vs. 600 mg bid 0%. The 600 mg bid regimen for this drug therapy seems to be useful as a therapeutic option because it significantly suppressed FVC reduction, but how to alleviate the hepatic impairment caused by this therapy is an open issue.

##### The Hepatobiliary Toxicity of BMS-986020

A paper by Gill et al. dealing with the mechanism for the hepatobiliary toxicity of BMS-986020 was published recently [149]. BMS-986020 was shown to inhibit bile acid (BA) efflux transporters BSEP, MRP3, and MRP4 as well as BA canalicular efflux. In human liver cells and bile duct cells, this drug inhibited the mitochondrial function. BMS-986278, another LAP1 antagonist, did not inhibit BA canalicular efflux. Therefore, the hepatobiliary toxicity of BMS-986020 does not seem to be associated with the LPA1-antagonizing activity.

##### BMS-986278

BMS-986278 is a second-generation LPA1 antagonist differing structurally from BMS-986020 [149]. In the structure-activity relationship (SAR) studies on LPA1, the discovery of BMS-986020 and subsequent modifications led to development of BMS-986278 as the final compound [150]. BMS-986278 is free of the efflux transporter inhibitory activity observed with BMS-986020 [149,151], and it did not cause hepatic impairment in the phase 1 study [152]. A phase 2 clinical trial on BMS-986278 (NCT04308681) was conducted in patients with IPF or PF-ILD [3]. The trial started on July 29, 2020 and was completed on August 4, 2022 (Actual Primary Completion Date). The trial was planned to enroll IPF patients (n = 240) and PF-ILD patients (n = 120), to be allocated at random to the BMS-986278 30 mg PO bid group, the BMS-986278 60 mg PO bid group, and the placebo PO bid group at a ratio of 1:1:1. The inclusion criteria were patients satisfying the diagnostic criteria for IPF or PF-ILD, with predicted %FVC ≥ 40%, a forced expiratory volume in 1 s (FEV1)/FVC ≥ 0.7 and DLCO ≥ 25%. The primary endpoint was the reduction rate in predicted %FVC [3]. Although the results of analysis from this trial have not yet been reported, effectiveness and tolerability in terms of adverse events are expected of this drug which manifests LPA1 inhibitory activity akin to BMS-986020 but is free of efflux transporter inhibitory activity which can lead to hepatic impairment (a problem with BMS-986020).

#### 4.4.2. ATX Inhibitors

Clinical studies on ATX inhibitors have been conducted on GLPG1690 and BBT-877.

##### Phase 2 FLORA Trial

GLPG1690 is the first ATX inhibitor used for IPF. Clinical trials on this drug have proceeded to the phase 3. Here, the phase 2 FLORA trial (NCT02738801) conducted previously is presented first. During the period from March 24, 2016, to May 2, 2017, screening was conducted on 72 patients and 23 of them were enrolled to the trial. Six patients were allocated to the placebo group and 17 patients to the GLPG1690 600 mg qd group, each planned to receive 12-week oral treatment according to the protocol [1]. The primary outcomes were safety (adverse events), tolerability, pharmacokinetics, and pharmacodynamics. Spirometry was assessed as a secondary outcome. The inclusion criteria were patients confirmed to have IPF by the central review, with FVC ≥ 50%, DLCO ≥ 30%, FEV1/FVC ≥ 0.70, and life expectancy ≥ 12 months. One patient in each group quit the trial prematurely because of adverse events and another patient in the GLPG1690 group cancelled consent to the trial. As a result, the data were analyzed on 5 patients in the placebo group and 15 patients in the GLPG1690 group [1]. Treatment-related adverse events, such as infection, developed in 4 patients (67%) from the placebo group and 11 patients (65%) from the GLPG1690 group, but most events were mild to moderate. Severe adverse events were recorded in 3 cases (2 cases from the placebo group and 1 case from the GLPG1690 group), including a case of urinary tract infection, acute nephropathy and lower airway infection, and a case of second-degree atrioventricular block from the placebo group as well as a case of cholangiocarcinoma from the GLPG1690 group. ATX activity was evaluated on the basis of changes in the serum LPA (18:2) level, revealing a reduction of the LPA level in the GLPG1690 group and improvement to the baseline level after discontinuation of treatment [1]. This result was close to the result of phase 1 clinical trials involving healthy volunteers (NCT02179502, NCT03143712) [153]. The mean FVC at Week 12, evaluated as a secondary outcome, was −70 mL in the placebo group and +25 mL in the GLPG1690 group [1].

##### Phase 3 ISABELA1 and ISABELA2

In the phase 2 trial, the safety of GLPG1690 comparable to a placebo was demonstrated, and the reduction in respiratory function at Week 12 (a secondary outcome) was also suppressed. On the basis of these results, two phase 3 clinical trials, i.e., ISABELA1 (NCT03711162) and ISABELA2 (NCT03733444), were started in November 2018 [154]. Both trials had an identical design, involving IPF patients (international, randomized, double-blind, placebo-controlled, parallel-group, multicenter studies). In each trial, 750 patients with IPF were allocated at a ratio of 1:1:1 to three groups (GLPG1690 600 mg po qd, GLPG1690 200 mg po qd, and placebo) in which GLPG1690 or a placebo was administered as an add-on to the standard therapy). The primary endpoint was the rate of decline of forced vital capacity (FVC) over 52 weeks. The trial was discontinued prematurely on the grounds that the benefit-risk profile no longer supported continuing the study based on recommendations of the Independent Data Monitoring Committee. The exact reason for its discontinuation is unknown, but the study data can be accessed at the U.S. National Library of Medicine, ClinicalTrials.gov. It seems that the all-cause mortality was probably related to the discontinuation of this trial.

##### BBT-877

BBT-877 is an ATX inhibitor on which antifibrotic activity and toxicity have been checked in animals [155]. On February 13, 2019, a phase 1 clinical trial (NCT03830125) on this drug in healthy adults was started. It was designed to allocate the subjects to a placebo group, a BBT-877 single dose group, and a BBT-877 multiple dose group. The trial was completed on November 24, 2019. The publication of its results is now anticipated.

## 5. Challenges and Future Scope

In recent years, mass spectrometry techniques have been widely used to identify lipids at the molecular level for samples derived from IPF patients. As mentioned above, lipid metabolites not only serve as bioactive substances, but also may have potential value as supplementary tests for IPF diagnosis [113]. In the field of regenerative medicine, lipids measuring may also enable quality control of regenerative medical products used in IPF [127]. Although how much contribution of lipid metabolism to the pathogenesis of IPF is controversial, at least clinical studies have shown that treatments targeting lipid metabolism (LPA1 antagonist, ATX inhibitor) for IPF have a reasonable effect [1,2]. We are hoping that the drug will be made available as a clinical use after further clinical trials.

Lipid-targeted IPF therapies may be differently affected for an individual lipid profile of each patient. For example, they may be more effective for patient groups in which LPA signaling is a major contributor to the pathophysiology, and vice versa. Measurement of patients’ BALF and serum lipid profiles might be useful as a predictor for therapeutic efficacy. When various targeted therapeutic agents were used in IPF, their combined efficacy would be examined. In addition, appropriate dosing of those drugs may be examined, and individualized treatment options may be selected based on background factors such as age, gender, and lipid profiles of patients in IPF.

## 6. Conclusions

This review paper has outlined the pathogenesis and treatment of IPF and summarized the involvement of lipids in the pathogenesis of IPF attracting close attention in recent years. The cascade related to LPA is now attracting particularly close attention, and the authors have described in this paper not only the involvement of LPA in IPF pathogenesis but also the clinical trials under way on this lipid. The pathogenesis of IPF is difficult to explain by a single mechanism. The disease develops under a complex involvement of chronic alveolar injury, genetic predisposition, and environmental factors. The pathogenesis of IPF involving lipid metabolism has been outlined in this paper, but the etiology of this disease can include diverse factors in addition to the factors mentioned above, e.g., regulatory T cells [156], ILC2 [157], and cell migration factors [158]. Because IPF is a disease with such heterogenous features, it is desirable to develop drugs with various treatment targets.

Inhibition of ATX or LPA receptors differs from the target of pirfenidone or nintedanib and may serve as a valid strategy for the treatment of IPF from now on. Because the LPA pathways are involved also in the homeostasis of organisms [159], treatment targeting the lipids (particularly the treatment inhibiting the metabolic enzymes such as ATX) can cause abnormalities in normal tissues. Therefore, when applying a therapeutic means targeting the ATX, special skills to enhance transfer to or accumulation in the lungs are needed, e.g., administering the drug by means of inhalation.

Although IPF is a disease with a poor prognosis, its pathogenesis has been increasingly unveiled in recent years, and there have been increasing efforts for developing novel methods of treatment. Hopefully, lipid metabolism in the mechanism of lung fibrosis will be further elucidated, and therapies targeting it will lead to real-world clinical practice and help overcome IPF.

## Figures and Tables

**Table 1:** Clinical trials on the LPA pathway for IPF. Clinical studies on LPA pathways related to IPF (revealed by search via the U.S. National Library of Medicine, ClinicalTrials.gov).

Drug Name	Mechanism	Trial Number	Phase	Disease	Status at Nov 2022
BMS-986020	LPA1 antagonist	NCT01766817	Phase II	IPF	Completed
BMS-986278	LPA1 antagonist	NCT04308681	Phase II	IPF and PF-ILD	Active, not recruiting
GLPG1690	ATX inhibitor	NCT02738801	Phase II	IPF	Completed
		NCT03711162	Phase III	IPF	Terminated
		NCT03733444	Phase III	IPF	Terminated
BBT-877	ATX inhibitor	NCT03830125	Phase I		Completed

## Data Availability

Not applicable.
